# State budget transfers to Health Insurance Funds for universal health coverage: institutional design patterns and challenges of covering those outside the formal sector in Eastern European high-income countries

**DOI:** 10.1186/s12939-016-0295-y

**Published:** 2016-01-15

**Authors:** Ileana Vilcu, Inke Mathauer

**Affiliations:** Consultant with the World Health Organization from October 2014 to December 2015, Avenue Appia, 1211 Geneva, Switzerland; Department of Health Systems Governance and Financing, World Health Organization, Avenue Appia, 1211 Geneva, Switzerland

**Keywords:** Universal health coverage, Vulnerable population groups outside formal sector work, Government subsidization of health insurance, State budget transfers, Financial protection

## Abstract

**Introduction:**

Many countries from the European region, which moved from a government financed and provided health system to social health insurance, would have had the risk of moving away from universal health coverage if they had followed a “traditional” approach. The Eastern European high-income countries studied in this paper managed to avoid this potential pitfall by using state budget revenues to explicitly pay health insurance contributions on behalf of certain (vulnerable) population groups who have difficulties to pay these contributions themselves.

The institutional design aspects of their government revenue transfer arrangements are analysed, as well as their impact on universal health coverage progress.

**Methods:**

This regional study is based on literature review and review of databases for the performance assessment. The analytical framework focuses on the following institutional design features: rules on eligibility for contribution exemption, financing and pooling arrangements, and purchasing arrangements and benefit package design.

**Results:**

More commonalities than differences can be identified across countries: a broad range of groups eligible for exemption from payment of health insurance contributions, full state contributions on behalf of the exempted groups, mostly mandatory participation, integrated pools for both the exempted and contributors, and relatively comprehensive benefit packages. In terms of performance, all countries have high total population coverage rates, but there are still challenges regarding financial protection and access to and utilization of health care services, especially for low income people.

**Conclusion:**

Overall, government revenue transfer arrangements to exempt vulnerable groups from contributions are one option to progress towards universal health coverage.

**Electronic supplementary material:**

The online version of this article (doi:10.1186/s12939-016-0295-y) contains supplementary material, which is available to authorized users.

## Introduction

Several countries from Central and Eastern Europe and the former Soviet Union moved from a government financed and provided health system (Semashko model) to social health insurance (SHI) and introduced payroll taxes in the early 1990s (the full list of abbreviations can be found at the beginning of the document) [1]. Prior to this transition, they were characterized by high levels of financial protection and equity in access [2]. This reform would have had the risk of moving away from universal population coverage if implementation had followed a “traditional” SHI approach that would focus on the formal sector employees and their dependents only. The countries covered in this paper mostly managed to avoid this potential pitfall as they transitioned to a contributory health insurance system [3]. In a “traditional” SHI, those not contributing are not covered by the SHI (and thus only access state provided health services). But certain population groups are too poor to pay insurance contributions themselves and they need to be exempted from payment of health insurance contribution in order to remain entitled for health services. Thus, the government budget was the starting point for health financing, and state budget transfers were built into the design of the introduction of earmarked payroll taxes right from the start in nearly all countries. In fact, an increasing number of governments across the globe decide to use general government revenues, i.e. state budget transfers, to explicitly pay the contributions on behalf of non-contributing population groups, most often in addition to cross-subsidization from within the fund’s contribution, because they realised that progress towards universal health coverage (UHC) is not possible via payroll taxes of contributors only. This arrangement is also frequently captured and termed as government subsidization of insurance contributions, usually for economically inactive, vulnerable and poor population groups depending on the context [3–7]. Vulnerable groups can be defined as “groups whose demographic, geographic, or economic characteristics impede or prevent their access to health care services” ([8], p. 253).

The specific focus on such budget transfer/subsidization arrangements via some form of health insurance, however, does not suggest that this financing arrangement is the only path to move towards or deepen UHC. One way is to expand user fee exemptions for specific groups, often also called free health-care policies for selected services and/or selected population groups, such as in Africa primarily. Other countries in Eastern and Central Europe and Central Asia have done so without payroll taxes, even though some of them established a separate purchasing agency, such as Latvia. These approaches tend not to be based on the affiliation and enrolment of entitled individuals. In the past, another group of countries tried to rely solely on payroll taxes and SHI to expand coverage without any state budget transfers. But a growing number of countries, both globally as well as in the European region, is applying a mix of contributory and non-contributory approach, by having chosen the path of government subsidization/government budget transfers to a health insurance type system. This deserves a specific analysis which is what this paper chose as its explicit focus.

The paper’s aim is to analyse the institutional design features of these government revenue transfer arrangements to SHI funds that serve to cover non-contributors in the health insurance systems, with a particular emphasis on those outside the formal sector, many of them being the poor and other vulnerable population groups, although it is difficult to separate these from other population groups that are also eligible for contribution exemption. For shortening purpose, from now on we refer to them as “vulnerable groups” or “the exempted”. The regional focus of this paper is on Eastern European high-income countries (as per World Bank country income classification [9]).

The existing body of literature contains single country studies, assessing SHI and government subsidization arrangements in particularly or discussing these as part of a broader health system review. Kutzin et al. [10] provide a comprehensive assessment of health financing reforms of former Eastern Bloc countries. Taking it from there, this paper explores in detail the specific institutional design features of SHI arrangements that incorporate government revenue transfer and assesses the progress and achievements or setbacks as to UHC. UHC means that “all people have access to services and do not suffer financial hardship paying for them” ([7], p. 9). The performance assessment also includes a comparison between contributors and the exempt population groups. As a result, those critical institutional design features that are particularly conductive for UHC progress are identified. This analysis reveals that these financing arrangements are indeed one option to progress towards UHC.

The remaining of the paper is structured as follows. The next section outlines the applied methods and describes the analytical framework to research institutional design, as well as UHC related indicators to assess the performance of these financing arrangements. Section 3 presents findings, which is followed by a discussion of potential effects of specific institutional design aspects as well as of challenges on progress towards UHC in Section 4. The last section concludes with policy lessons.

## Methods

This paper’s focus is on high-income Eastern European countries of the World Health Organization (WHO) European Region that introduced payroll taxation as a new earmarked tax for a new system of compulsory health insurance combined with government revenue transfers for non-contributors. These countries are Croatia, the Czech Republic, Estonia, Hungary, Poland, Slovakia and Slovenia, which were part of the former Eastern Bloc and which have turned into high-income countries over the past years, i.e. that were classified so for at least four consecutive years since 2008 [11]. This paper thus complements a paper by Mathauer et al. [12] of the same subject on low- and middle-income countries of the WHO European Region and is part of a series of regional studies and a global review all with the same research focus on state budget transfer/government subsidization arrangements.

To include or exclude countries in the study, in a first step, former Soviet and Eastern Bloc countries of the WHO European Region which have entered the group of high-income countries and for which the Global Health Expenditure Database indicated social security expenditure in 2012 were selected. In a second step, based on literature screening, countries were included if and when the SHI system was combined with government budget transfers for non-contributors. Once this list of countries was established, a detailed literature search was undertaken for each country in Science Direct, JSTOR, PubMed and Google. In Google, the first five pages, with ten results per page, were screened for publications and grey literature from universities, non-governmental organizations, governments, international organisations and alike. In the case of the other databases the first 100 findings were screened. In addition, the most recent Health Systems in Transition publications were used. For the institutional design analysis, the following search term combinations were used: health system OR health financing OR health insurance OR health vulnerable OR budget transfer OR government revenue transfer OR health subsid* AND the respective country AND/OR fund’s name.

The literature search for the performance assessment was based on the following search term combinations: health insurance coverage OR out-of-pocket payments (OOP) OR catastrophic health expenditure OR impoverish* health care OR financial protection OR access health OR utilization health care OR universal coverage AND the respective country AND/OR fund’s name. Furthermore, data was also collected from the WHO (for population size and OOP as a share of total health expenditure (THE)), Organization for Economic Co-operation and Development (OECD) (for the total population coverage rates) and Eurostat (for the share of unmet need) databases, as well as other country statistics.

The analytical framework used to assess institutional design of government subsidization arrangements is guided by the health financing functional approach of Kutzin [13] and looks specifically at the following aspects:

1. Revenue collection:
  Eligibility rules for exemption from contributions and targeting;
  Financing arrangements;
2. Pooling arrangements;
3. Purchasing arrangements and benefit package design.

The actual design features in place around these functions are the result of explicit or implicit policy choices.

Building upon the World Health Report’s UHC conceptualization, progress towards UHC is assessed by looking at changes and improvement in the following three indicators: 1) Population coverage, understood as affiliation to the health financing arrangement under study here; 2) financial protection; and 3) access to and utilization of needed health care services [14]. Table 1 outlines our framework and provides an overview of the institutional design aspects and related policy choices. It also shows how these potentially relate to and affect progress toward UHC. A detailed explanation of these institutional design aspects and indicators can be found in Additional file 1.

**Table 1:** Institutional design features of government revenue transfer/government subsidization

Institutional design aspect	Related policy choices	Intermediate output indicators	UHC related performance indicators
Eligibility and enrolment rules			
Groups eligible for exemption from contributions/subsidization	Definition of vulnerability (e.g. children, unemployed, pregnant women, informal sector workers, poor, near poor)	Share of the eligible among the bottom two income quintiles and other vulnerable groups	Total population coverage (comprehensiveness of the health insurance system), differentiated along income quintiles
Targeting method	E.g. universal (based on a very broad criterion such as residence or no employment in the formal sector), indirect (based on socio-demographic, socio-economic or geographic characteristics usually correlated with poverty and vulnerability), direct (through a means assessment or proxy means testing); different targeting approaches can be in place at the same time for different groups	Share of the exempted/subsidized within total (insured) population; Share of the exempted/subsidized among those being targeted for exemption/subsidization (targeting effectiveness of the system)	
Enrolment process	Active enrolment by the beneficiary or automatic enrolment by the authorities		
Organization responsible for identification of the exempted non-contributors/the subsidized	E.g., insurance company; central, regional, local government		
Type of enrolment/membership	Mandatory or voluntary		
Financing arrangements			
Degree of subsidization/co-contribution	Full or partial (a co-contribution is required)	Share of the exempted/subsidized within total (insured) population/those being targeted for subsidization (importance of government revenue)	
Type of transfer mechanism	Individual-based (a specific amount is being paid for each exempted individual) or lump-sum (a lump sum transfer for the entire exempted population is made)		
Calculation logic to determine the amount being transferred	E.g., based on regular contribution levels, minimum or average wages, specific percentage of the government budget, negotiated by the government	Sufficient funding for a comprehensive benefit package	Financial protection (incidence of catastrophic^a^/impoverishing health expenditure), also differentiated along income quintiles and other aspects; Access to services
		Level of cross-subsidization from contributions	
Source of government revenue transfers	E.g. general government revenues, earmarked government revenues, transfers from other health insurance funds or from contributors within the same pool (cross-subsidization), donor funding		
Pooling arrangements			
Type of pool(s) (general)	Single fund or multiple funds	Degree of fragmentation,	Equity in access;
Type of pool (exempted/subsidized)	Exempted/subsidized integrated into existing fund(s) or separate fund for the exempted/subsidized	Size and composition of pools,	Equity in financing;
Type of health insurance affiliation/membership of the contributors	Voluntary or mandatory	Level of cross-subsidization	Efficiency;
			Financial protection
Purchasing arrangements and benefit package design			
Range of services covered by the benefit package	E.g. comprehensive, inpatient focus, outpatient focus, pharmaceuticals, dental care, indirect costs (e.g. transportation)		Financial protection;
	Different or same package as contributors		Access (utilization rates);
			Equity in access
Degree of cost-sharing	Cost-sharing mechanisms (e.g., co-insurance, co-payment, deductible) and rates		
Provider payment mechanisms	Type of provider payment and rates	Efficiency	
	Same or different rules around provider payment		

^a^As per the WHO definition, catastrophic expenditure “occurs when a household’s total out-of-pocket health payments equal or exceed 40 % of household’s capacity to pay” ([46], p. 4)

Source of table: adapted from [47]

## Results

### Country and system overview

The analysis focuses on seven Eastern European countries, namely Croatia, the Czech Republic, Estonia, Hungary, Poland, Slovakia and Slovenia. Table 2 provides a summary overview of countries. Prior to the system transition of the 1990s, these countries were classified as middle-income countries. During the Soviet Union era, their health systems was based on the Semashko model [15–19] with heavy centralist planning and state owned and financed health facilities ensured high levels of financial protection and equity in access [2].

**Table 2:** Country overview

Country	Year of shift to high-income classification [11]	Population (2013, in millions) [48]	GDP per capita (2013, in current US$) [49]	Poverty headcount ratio (% of population, 2012)^a^ [49]	Year of introduction of (social) health insurance	Year when budget transfer to exempt those unable to contribute was introduced
Croatia	2008	4,3	13,597	19.5	1993 [20]	1993 [50]
Czech Republic	2006	10,5	19,858	8.6	1992 [51]	1992 [51]
Estonia	2006	1,3	18, 877	18.6	1992 [51]	1999 [16]
Hungary	2007	9,9	13,485	14.3	1990 [52]	1990 [52]
Poland	2009	38,5	13,653	17.3	1999 [51]	1999 [51]
Slovakia	2007	5,4	18.049	12.8	1994 [22]	1994 [22]
Slovenia	1997	2,1	23,295	14.5	1992 [23]	1992 [23]

Legend: *GDP* gross domestic product

^a^At national poverty line

As part of the transition and health financing reforms, and with the aim to increase resource mobilization as a reaction to economic and state budget challenges, most countries introduced (and a few re-established) payroll taxation for a new system of compulsory SHI. Croatia, Czech Republic, Hungary, Slovakia and Slovenia coupled this with government revenue transfers in the early or mid-1990s, and Poland and Estonia did so in 1999 [20]. In a way, the shift to a SHI system made entitlement in principle contingent upon contributions, or payment of these by the state on behalf of those exempted. Yet, this link between contribution on behalf of the state and entitlement is often less explicit, like in Hungary or Czech Republic [21].

In a first step, entitlement to the health insurance coverage is based on permanent residence (Czech Republic, Estonia, Poland, Slovakia and Slovenia) or personal identification card (Hungary). As a second condition, definition of specific groups eligible for exemption from contributions are specified by law [16–18, 22–24].

### Eligibility and targeting to identify the exempted

For the European region, it is important to specify the terminology of eligibility. Whereas in other regions, it is appropriate to talk of the “subsidized”, the European region requires some further terminological specification. As everybody was covered prior to the transition, the introduction of payroll taxes for formal sector employees and a contributory SHI system implied that various population groups needed to be *exempted* from contributions while still remaining entitled. The transfer of government revenues on behalf of and for the exempt has also led to the term of the “state insured”.

An overview of eligibility rules for those exempted from contributions in each country are provided in Table 3, revealing strong commonalities as well as some distinct differences in a few countries. The population group most frequently exempted are the economically inactive people [22]. The exempted or state insured need to be distinguished from the insured family dependents who are covered via their principal affiliate through the principle of cross-subsidization by contributors. Family dependents usually include non-working spouses and children. Yet, in Croatia, Czech Republic, Hungary and Poland, children are actually part of the state insured for which the government provides explicit budget transfers.

**Table 3:** Eligible groups for exemption from contribution

Country	Terminology used for eligible groups	Eligible groups for exemption from contributions		Family insurance in place	Type of membership of the exempt
Croatia	Vulnerable [50]	Unemployed;	War veterans;	Yes [50]	Mandatory [50]
		Disabled persons;	Militaries;		
		Children < 18 years;	Pensioners with pensions below the average net wage;		
		Students;	Farmers above age 66 [28, 29]		
Czech Republic	State insured [24]	Unemployed;	Women taking care of one child < 7 years or more children < 15 years;	Yes [24]	Mandatory [24]
		Pensioners;	Military personnel;		
		Children;	Prisoners;		
		Students;	People receiving social welfare;		
		Women or men on parental leave;	Asylum seekers [31]		
Estonia	Insured covered by state [16]	Individuals on parental leave with children < 3 years;	Individuals exposed to nuclear contamination, mainly related to the Chernobyl nuclear accident;	Yes [16]	Mandatory [16]
		One non-working parent of children < 8 years;	People receiving social benefits;		
		One parent in families with three children < 19 years;	Dependent spouses of diplomats;		
		Carers of disabled people;	Registered unemployed (entitlement for 270 days) [16]		
		Military personnel;			
		*Non-contributing insured people (almost half of insured people):* children up to 19 years, pensioners, disabled people entitled to special pensions, students, non-working spouses of insured individuals, non-working pregnant women from the 12th week of pregnancy [16]			
Hungary	Non-contributing groups [17]	Pensioners;	People with disability;	Yes [17]	Mandatory [52]
		Women on maternity leave;	Children < 18 years;		
		People with very low income (including homeless persons);	Students;		
		Military personnel;	Roma population [17, 43, 52]		
		The dependants of all the above;			
Poland	Non-contributing groups [18]	Children < 18 years;	People not eligible for unemployment benefits;	Yes [18]	Mandatory [18]
		Pregnant women;	People with low income;		
		Individuals with disabilities;	Soldiers;		
		People above age 65 without an old age or disability pension;	Farmers;		
			People on parental leave [18, 53]		
Slovakia	State insured [22]	Dependent children and their carers;	Reservists;	Yes [54]	Mandatory [22]
		Pensioners;	Unemployed;		
		Job applicants not receiving any allowance;	People on sick leave [22, 54]		
		Persons receiving disability benefits;			
Slovenia	No specific term [23]	Individuals without income;	Unregistered unemployed;	Yes [23]	Mandatory [23]
		Prisoners;	Recipients of social security allowances [23, 55]		
		War veterans;			

While the categories of exempt persons are diverse, the most common population groups include the unemployed, pensioners, people receiving social assistance benefits, the poor and children below 18 years. Even though military personnel, who are employed by the Ministry of Defence, may be not considered as vulnerable, they are exempted from contributions in several countries (Croatia, Czech Republic, Estonia and Hungary). Estonia stands out to some extent: in addition to the groups for whom the government transfers revenues to the insurance fund, several population groups do not contribute and for which there is no state budget transfer made to contribute on their behalf. These encompass children up to 19 years, students, non-working pregnant women, non-working spouses of insured individuals, pensioners and disabled people entitled to special pensions. As in Croatia prior to its 2002 reform as in many Western European countries, this reflects the idea of insurance coverage of family dependents being cross-subsidized by the contributors.

Table 4 provides an overview of the targeting and enrolment rules. Notably, most countries apply indirect targeting to identify eligible individuals for contribution exemption, based on socio-economic characteristics. Only Hungary and Poland use a direct targeting approach based on income means testing. The organization responsible for the identification of eligible population groups is most often the local government authority. Nonetheless, the identified eligible need to undertake active steps for their enrolment into the SHI in several countries.

**Table 4:** Targeting and enrolment rules

Country	Targeting method applied	Responsible organization for identifying the eligible	Initiation of enrolment process
Croatia	Indirect targeting [50]	Local and central government [29]	n/a
Czech Republic	Indirect targeting [24]	Central government [24]	Active enrolment by the beneficiary [24]
Estonia	Indirect targeting [16]	Regional government [16]	Automatic enrolment by authorities [16]
Hungary	Indirect targeting and direct targeting (means testing) of people with very low income [17]	Local government [17]	Active enrolment by the beneficiary [52]
Poland	Indirect targeting and direct targeting (means testing) of people with low income [18]	Local government [18]	n/a
Slovakia	Indirect targeting [22]	Local government [22]	Active enrolment by the beneficiary [22]
Slovenia	Indirect targeting [23]	Local government [23]	n/a

### Financing arrangements

Two key health expenditure indicators related to the financing arrangements are general government health expenditure (GGHE) as a share of THE and expenditure of SHI funds as a share of GGHE (Table 5).

**Table 5:** Financing arrangements and health expenditure indicators

Country	Degree of exemption	Logic/formula to calculate the transfer amount for the non-contributors	Transfer mechanism	Financing source of transfer	GGHE as % of THE [48]			Expenditures of social health insurance fund as % of GGHE^a^ [48]		
					1995	Year prior to intro-duction of govt budget transfers	2013	1995	Year prior to intro-duction of govt budget transfers	2013
Croatia	100 % [28]	Pensioners with pensions below the average net wage: 1 % of gross pension	Individual-based for pensioners and unemployed	General taxation for pensioners and unemployed;	86	n/a	80	95	n/a	94
				Central and local governments budget for the rest [28]						
		Unemployed: 5 % of unemployment benefit [28]	Lump sum for the rest [20]							
		The rest: lump sum decided upon in the budget-making process [20]								
Czech Republic	100 % [24]	Contribution rate of 13.5 % applied to 25 % of the average monthly wage two years prior to the current year [24]	Individual-based [24]	Central government budget [19]	91	n/a	83	84	n/a	93
Estonia	100 % [16]	For the registered unemployed: 13 % of an annually defined amount	Individual-based [16]	Unemployment insurance fund	90	86 (1998)	78	86 (1999)	n/a	87
		The rest: 13 % of an annually defined amount [16]		Central government budget [40]						
Hungary	100 % [17]	Not specified [20]	Lump sum [17]	Central government budget [17]	84	n/a	64	80	n/a	83
Poland	100 % [18]	Calculated on the basis of unemployment benefits [18]	Individual-based [18]	Central government budget [18]	73	65 (1998)	70	84 (1999)	0 (1998)	86
Slovakia	100 % [22]	4.78 % of national average wage [22]	Individual-based [22]	Central government budget [22]	89	n/a	70	96	n/a	90
Slovenia	100 % [23]	Fixed amount [23]	Individual-based [23]	Central government budget, municipalities [23]	78	n/a	72	94	n/a	93

Legend: *GGHE* general government health expenditure; *THE* total health expenditure

^a^This figure includes the government revenue transfers

GGHE as a share of THE is relatively high in 1995, but decreases in all countries, reaching the lowest level in Hungary at 64 % (from 84 % in 1995). The core purchaser of health services are the SHI agencies, as at least 80 % of GGHE is spent via them, and in three countries, this share is above 90 %. This share has mostly gone up, slightly though, in four of the seven countries, ranging from 83 % in Hungary to 94 % in Croatia. Importantly, this share includes the government revenue transfers. While precise data is only available for Hungary, Slovakia and Estonia, general government revenue transfer to the insurance funds do constitute an important share in all countries. In Hungary, the share of general government revenue as of total SHI funds is largest (above 50 %) [25], followed by Slovakia (34 % in 2011) [26]. It is important to note though that budget transfers are not necessarily linked only to finance the non-contributors, but may equally serve to provide funding to the whole pool of both contributors and non-contributors. In Hungary, for example, it is also a means to complement government taxation policy to reduce contribution rates for the contributors. Estonia is an exception again, as this share is approximately 5 % only (in 2012) [27], given that the non-contributors are largely cross-subsidized from contributors.

The institutional design features relating to the financing arrangements are presented in the first four columns of Table 5. Importantly, in contrast to other regions, all countries studied here fully exempt those considered as unable to pay contributions, i.e. eligible for exemption, and they do not co-contribute at all. This aspect is crucial, because the collection of partial contributions from those outside formal sector employment may be cumbersome and potentially hinder vulnerable groups to get enrolled.

However, some groups outside the formal sector are not eligible for exemption, i.e. no specific state budget transfers are made on their behalf. These groups have to make contributions and in some countries include the registered unemployed, farmers, craftsmen, pensioners and the self-employed.

Table 5 also presents the logic or formula of determining the amount of the state budget transfer for non-contributors. In all countries but two, the government transfer for the exempted is based on a per capita amount that is formula-based. One exception is Hungary, where the government transfers a lump sum that is largely a result of budget negotiation processes [17]. This is also the case for all non-contributors of Croatia except those who receive unemployment benefits or pensions below the average net wage.

Various types of calculation formula and logics are found. First, in the Czech Republic and Slovakia, the transfer amount is determined by applying a set rate to the average wage, or as in Estonia, on an annually defined amount. Yet, notably, in Slovakia, the contribution rate for the contributing population is 14 % [22], whereas a rate of 4.78 % is applied for the exempt groups, pointing to cross-subsidization from contributors to the exempt. Secondly, in Poland, Croatia and Estonia, for social security beneficiaries, such as pensioners and the unemployed, the transfer amount is calculated by applying a set rate to social security pension or unemployment benefits, even though the source of funds are central government revenues, except in Estonia. A third way is applied in Slovenia where a fixed amount per capita is in place.

In all these countries, the transfer amount for the state insured has hence little to do with the average contribution amount of the contributors. Yet, this is not the objective. Different contribution rates applied to different “contribution” bases for different population groups within the same country are thus largely the result of taxation policy considerations as well as feasibility concerns. Importantly, these calculation formula are not intended to reflect an actuarial-based contribution to cover the exempted average expenditure. Instead, the aim is to achieve a funding level, in combination with contributions that cover the costs of care. Thus, in some countries, government budget transfers serve to “subsidize” all insured, whether contributing or not.

Regarding the source of transfers, all countries make use of central government revenues. Only in Estonia are unemployment funds the source of funds transferred for the registered unemployed. Moreover, a few countries make use of hypothecated taxes: in Croatia, since 2008, car insurance tax and 32 % of revenues from excise taxes on tobacco are designated to health financing [28, 29]. Hungary also introduced earmarked taxes for health financing, such as the fat taxes levied on foods with high fat, sugar, salt and caffeine content [30].

### Pooling arrangements

Strong commonalities in institutional design are found with respect to pooling arrangements (Table 6). As the countries transitioned and shifted from a universalist Semashko system (covering all population groups) to a compulsory SHI system, the most crucial feature is that all countries built in universality and chose to establish an integrated fund for both the contributors and non-contributors with the aspiration and logic to again cover the total population. While this followed the pre-transition logic of coverage for all, setting up an integrated pool for all population groups is not self-evident, as evidence from other regions reveals.

**Table 6:** Pooling arrangements

Country	Single/multiple pool(s)	Integrated/separated pool for the exempt and contributors	Type of membership of contributors
Croatia	Single: Croatian Health Insurance Institute [56]	Integrated [56]	Mandatory [50]
Czech Republic	Multiple [24]	Integrated [24]	Mandatory [24]
Estonia	Single: Estonian Health Insurance Fund [57]	Integrated [57]	Mandatory
			Voluntary: residents who receive a pension from abroad, unregistered unemployed, students studying beyond normal length of study [16]
Hungary	Single: Health Insurance Fund, administrated by the National Health Insurance Fund Administration [17]	Integrated [17]	Mandatory [17]
Poland	Single: National Health Fund [53]	Integrated [18]	Mandatory
			Voluntary for employees on unpaid leave, persons engaged in certain types of contract work, volunteers [18]
Slovakia	Multiple [22]	Integrated [22]	Mandatory [22]
Slovenia	Single: Health Insurance Institute of Slovenia [23]	Integrated [23]	Mandatory [23]

This came along with another important policy choice, namely to introduce mandatory coverage for all - both contributors as well as non-contributors - except in Estonia and Poland, where a few smaller population groups can obtain insurance coverage on a voluntary basis.

Even though contributors and exempt are within the same pool, there is fragmentation in some countries. While Croatia, Estonia, Hungary, Poland and Slovenia have a single national pool, the Czech Republic and Slovakia introduced a multiple payer system at a later stage, with 9 funds [31] and 3 funds [22] respectively, combined with a risk equalization mechanism. In the Czech Republic, each health insurance fund collects the SHI contributions from all its members, or receives state budget transfers for the state insured. Individuals are free to enrol in any health insurance fund and they have the right to choose it once a year [24]. In 1994, the government introduced a risk adjustment mechanism to equalize available funds across insurers. The revenues subject to the risk adjustment mechanism comprised 100 % of state transfers on behalf of the state insured and 60 % of revenues from contributors. These funds were redistributed among insurers taking into account the number of state insured persons, further adjusted by two age categories (below and above 60 years). Those above 60 years of age are given a triple weight in the risk adjustment formula [32]. Although this approach allowed for more financial sustainability, it was ineffective because it did not eliminate incentives for risk selection [32]. Thus, in the late 2000s, the risk adjustment mechanisms was further modified, with 100 % of revenues from contributions being subject to redistribution and with a risk adjustment formula now based on age and gender, and no longer on exemption status. This further reduced incentives for risk selection [24].

In Slovakia, soon after the introduction of its SHI, competition between non-profit insurers was introduced [32]. Since 2010, after merging and various reforms in the legal status of the funds, the system ended up having three health insurance funds. Each collects SHI contributions and receives government revenue transfers, and individuals are free to enrol in any of them. A risk adjustment mechanism has been set up, initially based on an age- and gender-related risk index, which, since 2010, is applied in a differential way for the state insured and the contributors. The different risk-adjusted allocations for different population groups result in certain groups of the state insured being more attractive (e.g., students) than others (e.g., unemployed), possibly increasing incentives for risk selection by insurers [22].

### Purchasing arrangements and benefit package design

Since all countries operate integrated schemes for both contributors and the exempt, provider payment mechanisms and rates applied are the same for both groups, thus giving providers no immediate incentive for cream-skimming patients.

As Table 7 shows, in all the countries covered here, the SHI benefit package is relatively comprehensive and covered health services are the same for both the non-contributors and contributors. Where covered services offered at private health care providers are included, access for the state insured is the same as for contributors.

**Table 7:** Benefit package

Country	Range of services covered	
		Same/different compared to the contributors
Croatia	Comprehensive: primary care, inpatient and outpatient care, list of prescribed drugs, selected dental procedures [56]	Same [56]
Czech Republic	Comprehensive: outpatient and inpatient care, prescription drugs, selected drugs, rehabilitation, selected dental procedures, sanatoria treatment [24]	Same [24]
Estonia	Comprehensive: family doctor services, inpatient and outpatient specialist care, long-term care, rehabilitation, prescribed drugs [39]	Same [16]
Hungary	Comprehensive: primary care, secondary and tertiary care, drugs, selected dental care services [17]	Same [17]
Poland	Comprehensive: primary health care, outpatient specialist care, hospital treatment, psychiatric care and addiction treatment, certain dental care services, drugs [18]	Same [18]
Slovakia	Comprehensive: inpatient and outpatient care, selected drugs, basic dental care services [22]	n/a
Slovenia	Comprehensive: primary, secondary and tertiary services, drugs, medical devices, costs of travel to health facilities [23]	Same [23]

Although the range of covered health care benefits is broad in all countries, cost-sharing mechanisms are in place, with an overview provided in Table 8. However, most countries provide cost-sharing exemptions for some but not all state insured groups and non-contributors as well as some groups among the contributors.

**Table 8:** Cost-sharing arrangements

Country	Co-payments/coinsurance/benefit ceiling	Groups exempted from cost-sharing
Croatia	Co-payments for inpatient and outpatient hospital services (20 % of price), dental services (20 % of price), primary care, prescribed drugs	Children, pregnant women, people living below the poverty line are exempted from co-payments [28]
	Price cap for all co-payments [28]	
Czech Republic	Co-payments for dental care, medical aids, and some prescribed drugs	Children and adolescents up to the age of 18 years are exempted from user fees for doctor visits [24]
	User fees for doctor visits, hospitals stays, prescription drugs and the use of outpatient services outside the regular office hours (annual ceiling per insured individual)	
	Children and adolescents up to the age of 18 and people older than 65: lower annual ceiling [24]	
Estonia	Co-payments for outpatient specialist care (if contracted by health insurance), inpatient care, prescription drugs, prescribed drugs, dental care (except tooth preservation)	n/a
	Co-insurance for specific inpatient care services set by the Estonian Health Insurance Fund [16, 40]	
Hungary	Co-payments and co-insurance for drugs, medical aids and prostheses, balneotherapy, dental prostheses, treatment in sanatoria, long-term chronic care, some ‘hotel’ aspects of inpatient services	n/a
	Co-payments for non-referral specialist services, except in emergency cases; co-payments for services beyond the doctor’s recommended treatment [17]	
Poland	Cost-sharing for drugs, certain dental procedures and material, certain health resort services	Veterans with disabilities and their spouses if they are dependant, veterans’ widows or widowers if they are entitled to a survivor’s pension are exempted from co-payment [18]
	Co-payments for orthopaedic devices [18]	
Slovakia	User fees for prescriptions (drugs, medical devices) and various health services beyond primary and secondary outpatient care and inpatient care.	People with disabilities and children under 6 years are exempted from co-payments [54]
	Co-payments for drugs, sanatoria treatment and transport to health service [22]	
Slovenia	Co-payments for visits to GP, specialists, hospitals and laboratories for the use of services covered by the Health Insurance Institute of Slovenia [23]	Children, unemployed individuals, those with income below a certain threshold and chronically ill people are exempted from co-payments [23]

### Assessment of UHC related performance indicators

#### Population coverage

This section seeks to explore how countries fared on their path to universal health since they shifted to the hybrid financing system of SHI and state budget transfers. It starts by looking at total population enrolment rates. The population enrolled in SHI as a share of the whole population is above 99 % in 2 out of the 7 analysed countries (Czech Republic and Slovenia). For the other countries their population coverage rate is equally very high and above 90 % (see Table 9).

**Table 9:** Population coverage rates

Country	(Social) health insurance enrolment rateof total population^a^	Population groups among which some individuals are more likely not to be enrolled	Exempted as share of			Year
			Total population	Insured population	Eligible population	
Croatia	98.4 % [58]	n/a	64 %^b^ [28]	65%^b^	n/a	2008
Czech Republic	99.9 %^b^	Individuals from the Roma ethnic group [33]	58 % [19, 31]	58 % [19]	100 %	2011
Estonia	93.9 % [59]	Long-term unemployed	4.9 %^b^ (2011) [27]	5.3 %^b^ (2011) [27]	n/a	2014
		Men that do not belong to the economically active population between 30 and 50 years [36]				
Hungary	96.0 % [59]	Individuals from the Roma ethnic group [34]	n/a	n/a	n/a	2013
Poland	91.6 % [59]	Poor	n/a	n/a	n/a	2013
		Homeless				
		Children of uninsured parents				
		Youngsters kept in holding facilities [37]				
Slovakia	94.6 % [59]	Individuals from the Roma ethnic group [35]	61.5 %^b^ (2011) [26]	63.5 %^b^ (2011) [26]	n/a	2013
Slovenia	100 % [59]	n/a	n/a	n/a	n/a	2013

^a^Data taken from OECD, if not otherwise indicated

^b^Authors’ calculations based on data from countries’ Statistical Office or Health Insurance Fund reports

Data on the share of the exempt or state insured as of the total population is not available for all countries. Estimations suggest that the largest share is found in Croatia (64 %), followed by Slovakia (approx. 62 %) and the Czech Republic (58 %) [19]. Estonia has the lowest share of 4.9 %, but this needs to be seen in addition to the 45.8 % of the non-contributing population being cross-subsidized by contributors. These figures indicate the significance of exemption and non-contribution and the importance of government revenue transfers.

An important question is which type of people are not covered in the countries with a population coverage rate below 100 % and whether they fall outside the eligible groups for exemption or non-contribution. In fact, people of Roma ethnic groups may be most likely to remain uninsured. In 2011, 7 % of the Roma population in the Czech Republic was not covered by the SHI [33], 9 % in Hungary [34], and 3 % in Slovakia respectively [35]. However, these numbers are small in absolute terms considering that the Roma population represents approximately 3 % of the total population in the Czech Republic [33] and 2 % in Hungary and Slovakia [26].

In Estonia, the long-term unemployed and men that do not belong to the economically active population between 30 and 50 years old are not part of the defined eligible groups for non-contributions and are thus more likely not to be insured [36]. In the richest income quintile every tenth person was found to not have insurance, whereas the probability of being uninsured is four times higher among people in the lowest income quintile [16]. In Poland, approximately 1-2 % of the population, namely the very poor, are uninsured. However, since 2004, regardless of insurance status, very poor people as well as uninsured pregnant women and children below 18 years have access, though limited, to free publicly-financed health services [37].

Finally, a very small percentage of the population is not covered, because these individuals are insured in other EU Member States (e.g. in Slovakia, this is 2.4 % of population [22]).

#### Financial protection

Financial protection data that differentiates between contributors and non-contributors is not available. Therefore, this and the following sub-section also assess data differentiated along income quintiles, although non-contributors cannot be simply equated with low income quintile population groups. Nonetheless, looking at income quintile differences helps revealing inequities across the whole population.

Table 10 summarizes data on OOP expenditure. The OOP health expenditures as share of THE vary across the analysed countries, ranging from 12 % in Slovenia to 28 % in Hungary, which is very low in global comparison. Only in Poland did this share decrease, and in Croatia and Estonia only very marginally, since the introduction of the SHI system including its government revenue transfers. Data regarding the share of OPPs as household expenditure by income quintile or decile is only available for a few countries. In the Czech Republic, OOPs are low and distributed relatively even across household income deciles, in contrast to Estonia, Hungary and Slovakia, where financial protection of low income quintiles is a real concern.

**Table 10:** OOP expenditure

Country	OOPs as % of THE^a^ [48]		OOP expenditure as a share of household expenditure by income quintile/decile
	(in the year after the introduction of the government budget transfers)	(in 2013)	
Croatia	13.5 (1995)^b^	12.5	OOPs represent a heavy burden for some financially most vulnerable groups [50]
Czech Republic	5.2 (1993) [60]	15.7	Low OOPs distributed relatively evenly across household income decile [24]
Estonia	19.9 (2000)	18.9	People from lower quintiles spent proportionally more than those from higher quintiles. OOPs of 1st quintile almost exclusively spent on medicines. 5th quintile spent more on medicines and outpatient care.
			1st income quintile: households with individuals 65 years or older or with disabilities or chronic diseases face an increasing risk of relatively high expenditure [16]
Hungary	10.9 (1991) [52]	27.5	2008:
			1st income quintile: 7.3 % of income spent on OOPs (compared to 6.1 % in 2005)
			5th income quintile: 2.5 % of income spent on OPPs (compared to 2.2 % in 2005) [43]
Poland	29.9 (2000)	22.8	n/a
Slovakia	11.5 (1995)	22.1	Increase in OOPs due to user fee introduction and higher co-payments in 2003 affected the poor much more than the wealthy [22]
Slovenia	11.2 (1995)^c^	12.2	n/a

^a^Data taken from the Global Health Expenditure Database

^b^Data for 1994 was not found and the Global Health Expenditure Database provides data starting with 1995

^c^Data for 1993 was not found and the Global Health Expenditure Database provides data starting with 1995

Data to reveal changes and disaggregation in the incidence of catastrophic and impoverishing expenditure is scarce (Table 11). In Hungary, both indicators show a slight improvement, so does the incidence for impoverishing expenditure in Poland. In contrast, in the Czech Republic, incidence of catastrophic expenditure increased slightly, whereas in Estonia, it is fluctuating with no clear trend, while its incidence of impoverishing expenditure for the two bottom income quintiles seems to go down over the recent years.

**Table 11:** Incidence of catastrophic and impoverishing expenditure

Country	% of households faced with catastrophic expenditure	% of households faced with impoverishing expenditure
Croatia	2009: 7.6 % [61]	n/a
Czech Republic	1999: 0 % (this reflects the low levels of cost-sharing) [62]	n/a
	2007: 8.1 %	
	2008: 13 %	
	2009: 11.9 %	
	(at a threshold of 5 % of net income) [63]	
Estonia	2000-2007: Approx. 2-4 % [16]	2000: 3.7 %
	2009: 1.6 % [61]	2007: 2.1 % [36]
	Threshold of 40 %:	For the 1st quintile:
	2000: 1.8 %	2000: 4.6 %
	2001: 1.9 %	2001: 4.6 %
	2002: 2.1 %	2002: 5.7 %
	2003: 2.1 %	2003: 5.8 %
	2004: 3.0 %	2004: 8.4 %
	2005: 2.8 %	2005: 3.5 %
	2006: 4.4 %	2006: 7.8 %
	2007: 2.3 %	2007: 4.6 % [42]
	2010: 1.8 %	2010-2012 average for the 1st quintile: approx. 3 %
	2011: 1.4 %	2000-2007: approx. 5 % of single pensioners pushed below poverty line due to OOPs (compared to approx. 1 % in 2010–2012) [64]
	2012: 2.1 % [64]	
Hungary	2003: 0.7 %	2003: 0.2 %
	2007: 0.5 % [17]	2007: 0.1 % [17]
Poland	From 2000 to 2009: Incidence and intensity of catastrophic expenditure in drugs increased and affected for most the poor [65]	2000: 2.4 %
		2009: 1.4 %
	2009: 1.6 % [61]	63 % of the poor had drug expenditure and were further impoverished
		37 % of people fell into poverty due to drug expenditure [65]
Slovakia	Mean incidence of catastrophic health expenditure arising from OOPs: 0.6 % [66]	n/a
Slovenia	2009: 0.1 % [61]	n/a

### Access to and utilization of health care services

Table 12 provides data on utilization rates of health services across countries. Utilization rates of general practitioners (GP) services are equitable along income quintiles in 3 out of 7 countries (Czech Republic, Hungary and Slovenia). With respect to dental care, utilization rates are equitable in the Czech Republic and Slovenia, while in Croatia, Estonia, Hungary and Poland there is some inequity, with high-income people being advantaged.

**Table 12:** Utilization rates and unmet needs

Country	Equity between income quintiles	Inequities for lower income quintiles	Inequities for specific groups
Croatia	n/a	Lower utilization rates for GP, specialist and dentist visits [44]	n/a
		Higher share of unmet need^a^ (2012, [38])	
Czech Republic	Similar utilization rates for GP, specialist and dentist visits^b^ (2008, [67])	Higher share of unmet need^a^ (2012, [38])	44 % of Roma and 11 % of non-Roma population had no access to essential drugs. 87 % of Roma and 99 % of non-Roma population had access to health services (2011, [33])
Estonia	n/a	Inequities in access to primary and dental care, but declining (2004–2008, [36])	n/a
		Higher share of unmet need^a^ (2012, [38])	
		For services requiring user charges (outpatient drugs, dental care) there are more inequalities in utilization by income level compared to services with little need for OOPs (inpatient care, emergency care) (2000–2007, [36])	
Hungary	Equity in the probability of seeing a GP (2009, [67])	Inequity in utilization rates for dentist and specialist visits^b^ (2009, [67])	Roma were less likely to use health services, particularly those offered by specialist and dentists. The use of health services by Roma was similar to that seen in the lowest income quintile of the general population. (2007, [17])
		Higher share of unmet need^a^ (2012, [38])	
Poland	n/a	Higher share of unmet need^a^ (2012, [38])	n/a
		Inequity in utilization rates for GP and dentist visits^b^ (2009, [67])	
Slovakia	n/a	Inequity in utilization rates for GP visits^b^ (2009, [67])	n/a
		Higher share of unmet need^a^ (2012, [38])	
Slovenia	Equity in access and utilization rates [23]	n/a	n/a
	Equity in utilization rates for GP, specialist and dentist visits^b^ (2007, [67])		

^a^Information regarding the share of unmet need is from Eurostat

^b^Data on utilization of health care services by income level is adjusted for need

In 2012, in all countries, except Slovenia where data is not available, within the poorest income quintile, the percentage of people reporting unmet needs for medical examination is higher in comparison to better-off people. Among the reasons, unaffordability (too expensive), too long distance to travel or long waiting lists are mentioned [38].

In the Czech Republic and Hungary, when it comes to utilization rates and access to health services of specific population groups, the Roma are disadvantaged compared to non-Roma (see last column of Table 12). Moreover, in Poland, the poorest uninsured people receive mostly inpatient care and they have limited access to primary care or dental services in practice [37].

## Discussion

This section examines the plausible effects of the institutional design features on performance, i.e. progress towards UHC.

### Effects of eligibility and targeting

Overall, all countries have both a very high total population enrolment rate as well as a high share of the exempt and non-contributors. These countries achieve practically universality in population coverage. This is despite the fact that there are large shares of the population outside formal sector employment, which in the logic of a “traditional” compulsory SHI would be the principle entry point for coverage. Yet, eligibility of the exempt is defined both broadly as well as specifically to capture those outside formal sector employment. High population coverage rates are effectively achieved by primarily applying an indirect targeting approach. Most important seems the inclusion of the (unregistered) unemployed, which captures a large part of those outside formal sector employment. However this is not the case in Poland, Slovenia and Estonia for the long-term employed. On the other hand, these countries have not chosen to "automatically" enrol all those outside the formal sector, possibly to avoid informality becoming attractive. As a result, population coverage is not 100 % in that some few population groups and individuals remain uncovered. In sum, the specific definition of eligible groups for exemption is decisive for the level of population coverage. Interestingly, in most countries (Croatia, Czech Republic, Hungary, Poland, Slovakia), children are part of the state insured and their coverage is financed through government budget transfers, even though family insurance is in place in all the 7 countries studied here. This reduces in principle the degree of cross-subsidization among contributors. It also further blurs the line between coverage of family dependents and otherwise vulnerable population groups financed through government budget transfers.

Yet, there are gaps in health insurance coverage of certain groups, and thus countries are undertaking efforts to reach these unreached groups. In Estonia, for example, since the end of 2002, individuals who might otherwise remain uninsured can acquire voluntary coverage when meeting the following eligibility criteria: Estonian residents who receive a pension fund from another country and individuals who are not currently eligible, but who have been members of the Estonian Health Insurance Fund for at least 12 months in the two years prior to applying for voluntary membership, as well as their dependents (students and people temporarily out of work but not registered as unemployed) [16].

In Poland, although people with low income are eligible for exemption, there are still uninsured poor individuals [37]. A reason for this might be the income threshold set by the authorities to establish the eligibility of individuals to be exempt. Despite being eligible, some groups such as the children of uninsured parents and homeless were not eligible for mandatory membership and did not have the capacity to pay contributions to join the system voluntarily before 2007. Therefore, decision makers tried to identify those population groups of excluded people in order to provide access to care and coverage to them.

### Effects of financing and pooling arrangements

The analysed health systems are strongly based on solidarity. Importantly, all countries have avoided fragmentation and allow for risk pooling by having established an integrated fund for both the exempted and the contributors. Moreover, the levels of state budget transfers are substantial in all countries, and found to be relatively high in Hungary and Slovakia for which data is available. State budget transfers are calculated on the basis of minimum wage, average wage or unemployment benefits or another defined amount, unless they represent a fixed amount of the budget. In general, unemployment benefits or the minimum wage are much smaller than the average wage, and thus using the average wage as assessment base leads to higher per capita budget transfers and potentially lower internal cross-subsidization. This also determines the level of equity in financing as well as the fund’s financial sustainability.

For example, in Slovakia, state budget transfers amounted to about one third of total revenues of the SHI system, while about two thirds of the population are exempted (data for 2009, [22]). The Slovak SHI system is thus strongly based on cross-subsidization of contributors. Likewise in Estonia, there is a very high level of cross-subsidization within the pool where almost half of the insured population contribute, whereas state budget transfers are made for around 5 % of the population, whilst the rest of the insured is cross-subsidized from within the pool, with everybody being entitled to the same benefit package [39]. Thus, in the long run, the system’s financial fairness and sustainability might be threatened since the revenues largely come from contributions of the working population. In addition, the population is ageing and the share of working age individuals is decreasing [16]. Despite the recommendations by the Estonian Health Insurance Fund and WHO’s specialists in 2009 to extend the public revenue base, i.e. apply the social tax to non-wage income and to introduce government budget transfers on behalf of pensioners [40], the Estonian Government did not choose to implement this reform option [41].

### Effects of benefit package design

The benefit package design and, equally important, the cost-sharing mechanisms determine the degree of financial protection and utilization of health care services. In fact, in most countries (Croatia, Estonia, Hungary, Poland and Slovakia), there is inequity in access for lower income quintiles. In all of these countries, this is a result of unaffordable co-payments, especially for prescribed drugs, with the low income people being the most affected [18, 22, 36, 42–44].

After the transition and the shift to the new financing arrangement, combining SHI contributions and state budget transfers, OOPs increased in the Czech Republic, Hungary, Slovakia and Slovenia (Table 10) as a result of high utilization or overutilization of health care services [16, 17, 22, 24]. Hungary and Slovakia tried to manage utilization by introducing cost-sharing mechanisms [17, 22], but they were not successful in reducing over-utilization. In contrast Slovenia increased co-payments with only a small impact on access to health care and financial protection [21, 23], moreover since a policy on voluntary health insurance helped to cover user charges [45].

In Poland, uninsured poor individuals have free access to publicly financed services, yet this does not solve the challenge that poor people have no means to buy drugs for example, and thus, even if not always necessary, they prefer hospitalization care, where drugs are included.

## Conclusions

This paper explored the trends and patterns of institutional design aspects of the existing financing arrangements, that is the mix of compulsory SHI contributions and state budget transfers with a focus on population groups outside formal sector employment and especially vulnerable population groups. Notably, the analysis revealed more commonalities than differences across countries. First of all, in all countries, vulnerable groups are fully exempted from paying contributions. The main challenge here is to ensure that the amount of transfers in combination with payroll contributions as a function of taxation policy are sufficient and allow for sustainability of the health insurance system in the long run.

Second, all countries avoided fragmentation by having established integrated pools for both the exempted and contributors. This allowed for risk pooling and strong redistributive capacity. Additionally, coverage is mandatory for both the exempted and the contributors with no opt out option of the system, with the exception of a few contributing groups in Estonia and Poland.

Third, the benefit packages are relatively comprehensive and, more importantly, all insured individuals, exempted as well as contributing, are entitled to the same benefit package. In most countries, this is combined with exemptions from cost-sharing for several, though not all, exempted groups. Concerns, though, remain for some of the vulnerable groups specifically that are not exempted from cost-sharing, and options need to be found such that they do not face difficulties in access to and utilization of health care services.

In terms of progress towards UHC, all countries have a very high total population enrolment rate (above 90 %). Yet, there are still groups not eligible for exemption and immediate measures within the same exemption logic should be taken in this regard. There are also challenges with respect to financial protection, because in half of the countries (Croatia, Estonia, Hungary, Slovakia), lower income quintiles face a heavy burden of OOPs.

Further research should be conducted to analyse the impact of these arrangements with respect to UHC progress on the basis of which to explore options how to improve coverage, especially for vulnerable groups. Governments should take further steps in better defining, identifying and exempting the potential vulnerable groups (e.g. the very poor, unregistered unemployed). Another question is how they can address the issue of system sustainability. Moreover, differentiated data for the exempt versus contributors, insured versus uninsured, and across income quintiles is needed. Providing answers to all these questions may help countries to deepen their achievements in UHC.

Overall, this analysis suggests that the government revenue transfer arrangements for selected and in particular vulnerable groups who are exempt from contributions, have been an effective way to set up a social health insurance that avoided coverage gaps for those outside the formal sector. This mixed financing system has thus made it possible to largely maintain their population coverage level after the system transition. Together with country experiences from other regions, it confirms that this approach of exempting vulnerable groups from contributions, or as termed elsewhere their subsidization, is one option to progress towards universal health coverage, contingent upon conducive institutional design.

## Additional file

Additional file 1:
**Analytical Framework.** Provides a detailed explanation of Table 1 based on which the analytical framework was developed [13, 14, 20, 21, 46, 68–73]. (PDF 35 kb)

## Abbreviations

GDP: gross domestic product
GGHE: general government health expenditure
GP: general practitioners
OECD: Organization for Economic Co-operation and Development
OOP: out-of-pocket payments
SHI: social health insurance
THE: total health expenditure
UHC: universal health coverage
WHO: World Health Organization
