# Erratum to: State budget transfers to Health Insurance Funds for universal health coverage: institutional design patterns and challenges of covering those outside the formal sector in Eastern European high-income countries

**DOI:** 10.1186/s12939-016-0333-9

**Published:** 2016-03-07

**Authors:** World Health Organization, Ileana Vilcu, Inke Mathauer

**Affiliations:** Consultant with the World Health Organization from October 2014 to December 2015, Avenue Appia, 1211 Geneva, Switzerland; Department of Health Systems Governance and Financing, World Health Organization, Avenue Appia, 1211 Geneva, Switzerland

Unfortunately, after publication of this article [1] it was noticed that the copyright statement is incorrect. The corrected copyright statement can be seen below:

© 2016 World Health Organization; licensee BioMed Central. This is an open access article distributed under the terms of the Creative Commons Attribution IGO License (http://creativecommons.org/licenses/by/3.0/igo/legalcode), which permits unrestricted use, distribution, and reproduction in any medium, provided the original work is properly cited. In any reproduction of this article there should not be any suggestion that WHO or this article endorse any specific organisation or products. The use of the WHO logo is not permitted.

This notice should be preserved along with the article's original URL
