# Supplementary material for: State budget transfers to Health Insurance Funds for universal health coverage: institutional design patterns and challenges of covering those outside the formal sector in Eastern European high-income countries
Source: Int J Equity Health. 2016 Jan 15;15:7. doi: 10.1186/s12939-016-0295-y (PMC4714511; doi:10.1186/s12939-016-0295-y)
Supplement: Additional file 1: — Analytical Framework. Provides a detailed explanation of Table 1 based on which the analytical framework was developed [13, 14, 20, 21, 46, 68–73]. (PDF 35 kb) [file 12939_2016_295_MOESM1_ESM.pdf]

Additional File 1. Analytical Framework

Rules on eligibility for contribution exemptions

Eligibility rules are country-specific and determine which population groups are eligible for exemption of contribution. These are usually vulnerable people, but other population groups may also be exempted (68).

There are various methods of identifying eligible groups, such as direct targeting, indirect targeting, self-targeting or universal targeting. Direct targeting (or individual targeting) implies identification through a means assessment (68), such as means testing or "proxy means testing" (69). In this case an income threshold is necessary to establish who is eligible for exemption from contributions (68). Indirect targeting (or categorical targeting) is based on broader, socio-demographic, socio-economic or geographic characteristics usually correlated with poverty and vulnerability (69, 70). Finally, in the case of a universalist approach of eligibility, eligible individuals satisfy a very broad criterion, for example permanent residency in the respective country or working outside the formal sector. Usually, the different types of targeting are not mutually exclusive, i.e. two or more methods can be simultaneously employed to identify different groups (69).

Different organizations are responsible for identifying the eligible groups such as the central/regional/local government authorities or the insurance agencies themselves. Enrolment can be undertaken either by the responsible authorities or else the beneficiary needs to take active steps to enrol. Another key design feature is the type of membership of the exempted individuals, which can be mandatory or voluntary (68). All these aspects

related to the eligibility rules assumingly influence the population coverage rate of the exempted.

Financing arrangements

Different sources to pay on behalf of the non-contributors are used, such as the central/regional/local government budget or pension and unemployment related social security funds (20). Government revenues may also be earmarked for this purpose. Another important institutional design feature is the type of transfer mechanism. One option consists in a specific amount being paid on behalf of each exempted individual (individual-based). Alternatively, a transfer for the entire exempted population is made (lump sum transfer). Various calculation logics can be applied to determine the amount of transfers.

In addition to state budget transfers, there is always a degree of cross-subsidization both among the contributors (from the healthy to the sick and the better-off to the poorer ones), as well as between contributors and non-contributors. While important to recognize and capture, this is difficult to measure in the absence of average expenditure per capita for different health risk groups. Finally, another important design features is whether exemption from contributions is complete or partial – partial meaning that some share of the contribution needs to be paid by the individual.

Pooling arrangements

Pooling "refers to the accumulation of prepaid health care revenues on behalf of a population" ((13), p. 177), whereby financial resources are allocated from collection agencies to a single pool or multiple pools (71). One critical institutional design feature to explore is whether the pool for the exempt/the subsidized is separated or integrated with the pool for

contributors. This pooling architecture and related pooling rules determine the level of risk-pooling and fragmentation as well as the scope of cross-subsidization within or across pools (72, 73). These institutional design features affect financial protection as well as the level of equity in financing and access and the efficiency of the SHI system.

Purchasing arrangements

Purchasing refers to "the transfer of pooled resources to service providers on behalf of the population" ((13), p. 180). One core institutional design aspect related to purchasing is the benefit package for the exempted non-contributors. Key features are the range of services covered by the benefit package and the cost-sharing mechanisms and levels (21). A particular question is whether the exempted enjoy the same range of services and the same level of cost-sharing as the contributors. A more comprehensive benefit package would include emergency care, primary care, specialized outpatient care and inpatient care as well as essential medicines (68). These institutional design features influence utilization rates and hence the level of equity in access as well as the extent of financial protection.

A second set of key institutional design aspects relates to the type of provider payment mechanisms and rates in place. Of particular interest is whether payment mechanisms and/or rates are different for the exempted in comparison to the contributors, and if so what implications this has on health services access and the delivery of health services.

Population coverage

Population coverage by a subsidization arrangement can be measured in various ways. One is to look at the population covered by SHI as a share of the whole population, which will reveal the comprehensiveness of the health insurance system. A second one is to consider the

exempted individuals as a share of total population as well as the total insured population. This gives an idea of the importance of government revenue transfers. Finally, looking at the exempted as a ratio of theoretically eligible population, helps revealing the targeting effectiveness of the system.

Financial protection

Financial protection means that "people should not become poor as a result of using health care, nor should they be forced to choose between their physical (and mental) health and their economic well-being" ((71), p.7). There are different measures of financial protection. One is the incidence of catastrophic expenditure. As per the WHO definition, catastrophic expenditure "occurs when a household's total out-of-pocket health payments equal or exceed 40% of household's capacity to pay" ((46), p. 4). Other (lower) thresholds are also being applied in the literature. Another measure is the incidence of impoverishing expenditure, defined as follows: a non-poor household is impoverished by health payments when it becomes poor after paying for health services" ((46), p. 5). Data on OOP health expenditure as a share of household expenditure, when differentiated along different equity dimensions, is useful to assess the impact of state budget transfer/government subsidization arrangements.

Access to and utilization of needed health care services

Since one of the goals of UHC is to reduce the gap between need and utilization (14), it is important to assess whether people have effective coverage of needed health services. Data on health care needs is required to measure effective coverage and access to needed health services. In the absence of this data, utilization rates for inpatient and outpatient care are therefore used as proxy indicators.

Ideally, available data for these indicators is differentiated along various equity dimensions, in particular along income quintiles, gender, rural/urban divisions or along ethnic group affiliation, and for the purpose of this paper, along the exempted versus the contributors. To assess the impact of government revenue transfers as a means to provide insurance coverage to the exempted, time series for these indicators are needed.
